# Effectiveness of emergency medicine in longitudinal integrated clerkships

**DOI:** 10.3402/meo.v19.25429

**Published:** 2014-09-15

**Authors:** Kenny Banh, Rene Ramirez, Christina Thabit

**Affiliations:** 1 Department of Emergency Medicine, UCSF Fresno, Fresno, CA, USA; 2 UC Davis School of Medicine, Sacramento, CA, USA

**Keywords:** undifferentiated patient, assessment, management, longitudinal, emergency medicine

## Abstract

**Objective:**

This study investigated third-year students’ experience with the emergency medicine (EM) component in integrated longitudinal programs. The study aimed to see if EM could be integrated into third-year integrated longitudinal programs while addressing accreditation standards and increasing interest in EM.

**Methods:**

The authors surveyed students who participated in an integrated longitudinal program at University of California San Francisco School of Medicine (UCSF) from 2010 to 2012. The survey focused on four areas of EM: fit within an integrated longitudinal program; development of critical decision-making and judgment skills; development of differential diagnoses and treatment plans; increased interest in pursuing EM.

**Results:**

Overall, students thought that EM fits well with the goals of an integrated longitudinal curriculum. They also thought that it helped them develop their decision-making, clinical judgment, differential diagnoses, and treatment plans. There was also an increased interest in pursuing EM as a career option because of the EM component.

**Conclusions:**

EM can be well integrated into a third-year longitudinal curriculum. The undifferentiated patient work-up helps students develop critical skills in assessment and management. The lack of continuity did not interfere with the integrated longitudinal curriculum, instead the experience enhanced it.

Concerns have been raised about the quality of learning experiences afforded to medical students in traditional block clerkships. Attempting to address some of the educationally problematic structures in clinical training, a national call to transform undergraduate medical education was made (1–7). One is the fragmented nature of student experiences characteristic of brief rotations through various specialties, in which there is little opportunity to follow patients or establish long-term relationships with faculty members (3–10). To reduce this fragmentation, Hirsh and colleagues proposed a framework for organizing the third-year medical school curricula around continuity – specifically, continuity of curricula, patient care experiences, and relationships with teachers and supervisors (11). One way to achieve such continuity is to include longitudinal experiences in selected outpatient settings. In theory, these experiences can provide insight into patients’ progression through illness and health, and to build relationships with faculty that are more conducive to formative feedback and learner-centered instruction. Clinical experiences organized in this way can significantly improve the quality of learning processes and outcomes.

Several US medical schools have added longitudinal components to their curricula, typically one half-day per week spent in clinic with a supervising doctor. Only a few medical schools, however, have implemented completely integrated longitudinal models. Started in 2004, outcomes data from Harvard Medical School's Cambridge Integrated Clerkship (HMS-CIC) have been encouraging (12). In this model, students see patients more frequently before a diagnosis is made and after discharge from the hospital; moreover, they are supervised by experienced faculty, rather than residents, to a much greater extent. In tests of knowledge and clinical skills, these students performed at least as well on exams of clinical knowledge – but felt better prepared with patient-centered aspects of care (13). At the same time, clinical preceptors and tutorial facilitators are enthusiastic about teaching – some, for the first time after many years of frustration (14, 15).

A similar year-long program, known as the Parnassus Integrated Student Clinical Experiences (PISCES), was implemented at the University of California, San Francisco (UCSF) Medical School in 2008. It has since spawned two other integrated longitudinal curricula at UCSF: the Kaiser Longitudinal Integrated Curriculum (KLIC), and the Longitudinal Integrated Fresno Experience (LIFE) – with the latter being located at affiliate hospitals (Kaiser-Oakland and UCSF-Fresno).

Meanwhile, the Liaison Committee for Medical Education (LCME), the primary accrediting body for US and Canadian allopathic training programs, recently updated its guidelines to include multidisciplinary clinical training opportunities that extend beyond traditional third-year clerkships, including emergency medicine (EM), geriatrics, and neurology. Specific EM core skills were also among these 2010 updates. These include the ability to develop critical judgment skills and interpret signs and symptoms to develop differential diagnoses and treatment plans.

Still, few medical schools require a third-year EM rotation, (16) and no integrated longitudinal programs outside of UCSF feature an EM component. Where, besides taking call on internal and family medicine, students also train in the high-acuity emergency department. While it may seem odd to facilitate continuity of care in an EM experience, using emergency physicians to illustrate patients’ presentation of undifferentiated complaints through admission, discharge, and clinic follow-up seemed a logical gateway to patients’ initial access of care.

## Methods

E-mail questionnaires, with weekly reminders, were sent out to UCSF medical students completing a third-year integrated longitudinal rotation at UCSF-Fresno, Kaiser-Oakland, or UCSF-Parnassus during AY2010–11 and AY2011–12. LIFE is a 6-month program with nine students per academic year while KLIC and PISCES are year-long programs with 8 and 16 students, respectively.

## Results

Overall, 44 of 56 (78%) students responded to the survey – which included several 5-point, Likert-type items ranging from ‘Strongly Disagree’ (1) to ‘Strongly Agree’ (5). Students overwhelmingly thought that their EM component was consistent with their longitudinal program goals (*M*=4.07) and helped them develop critical judgment (*M*=4.36) and differential diagnosis/treatment plans (*M*=4.70). Students’ interests in pursuing EM as a career (*M*=3.41) were also significantly positive but to a lesser extent.

## Discussion

Longitudinal medical curricula allow trainees to experience the natural progression of patients’ medical work-ups. As more medical schools adopt longitudinal approaches, EM could be integral in meeting the goals of such endeavors. The emergency department is an ideal place for students to encounter an undifferentiated patient, follow them during their hospital course to the point of discharge, and back again during outpatient clinic follow-up. Because the LIFE curriculum includes family medicine, internal medicine, neurology, and psychiatry, students are afforded the ability to reinforce educational objectives pertaining to any of these clerkships.

The EM curricular component is careful not to belabor students with stringent procedural or patient encounter requirements, and students’ interest and competency levels allow the freedom to pursue self-directed learning relative to encounters and procedures. With the guidance from EM faculty familiar with the learning objectives of the program, the student can readily apply classroom knowledge to critical decision-making, differential diagnosis, and treatment plans. The confidence gained by working up a patient, without an initial history or complete physical exam, is often what students find most beneficial to their medical training. With the integration of mainstream electronic medical records, patients could seamlessly transition to appropriate outpatient clinic follow-ups with the same medical student they encountered in the emergency department. From this initial encounter to the outpatient clinic, this makes for a meaningful experience of longitudinal care and patient continuity.
